# Supplementary material for: Convergent direct and indirect cortical streams shape avoidance decisions in mice via the midline thalamus
Source: Nat Commun. 2024 Aug 4;15:6598. doi: 10.1038/s41467-024-50941-6 (PMC11297946; doi:10.1038/s41467-024-50941-6)
Supplement: Supplementary file 1 — Supplementary Information [file 41467_2024_50941_MOESM1_ESM.pdf]

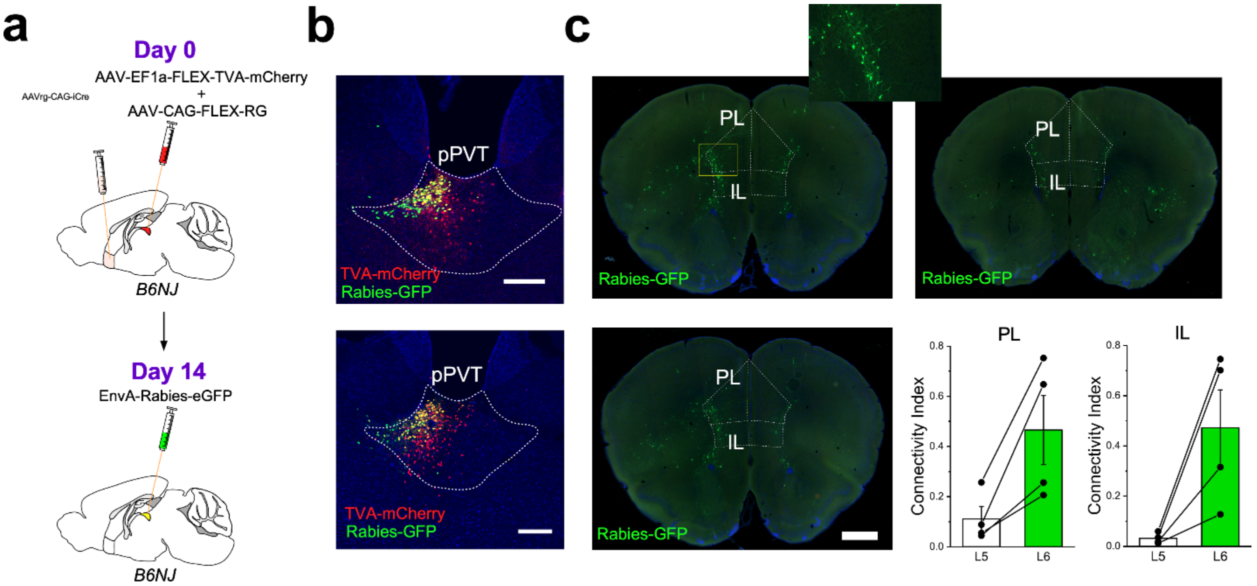

Supplementary Figure 1. Rabies-assisted mapping of layer specific mPFC projections to pPVT.

a, Schematic of the experimental approach for tracing monosynaptic inputs onto NAc–projectors of the pPVT. **b**, Representative images showing the rabies starter cells (GFP^+ and $mCherry^+$ double-labelled cells)(Scale bar: 200 μm). **c**, Representative images showing retrogradely-labeled (GFP^+) cells in the mPFC. Average cell counts for layer 5 and 6 of both the PL and IL are presented (n =12 slices from 4 mice) (Scale bar: 2 mm). PL: Independent two-tailed *t*-test, $t_{(3)} = 3.493$, $^*P = 0.0397$. IL: Independent two-tailed *t*-test, $t_{(3)}=3.136$, $P=0.0518$. Data are shown as mean ± s.e.m. Source data are provided as a Source Data file.

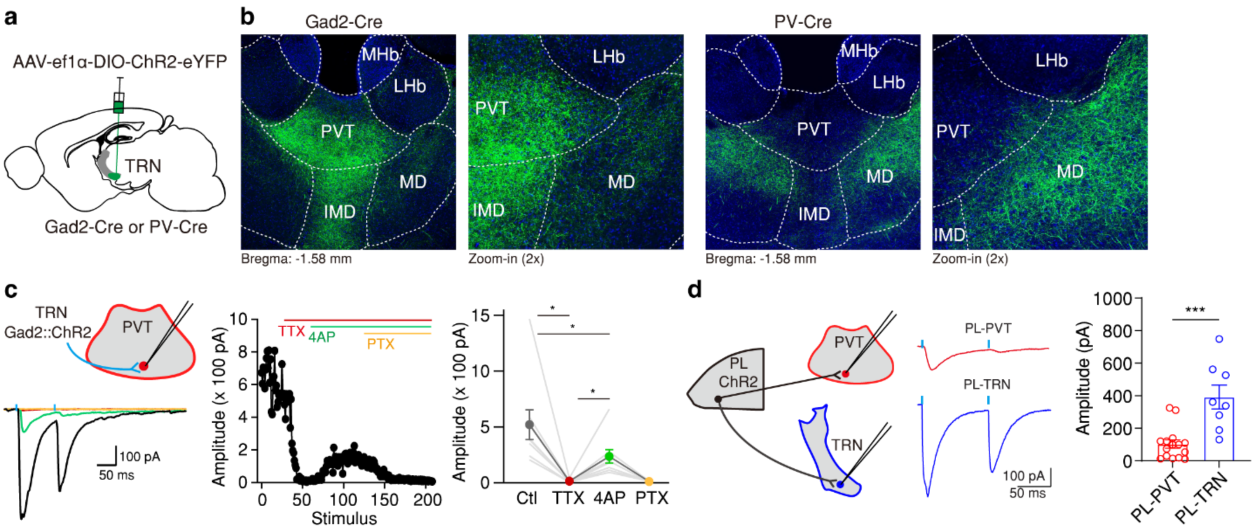

Supplementary Figure 2. Anatomical and electrophysiological characterization of avTRN projections to the PVT.

a, Schematic of the viral vector strategy used for anterograde tracing of the anatomical projections of GAD2^{+} (Gad2-Cre) and PV^{+} (PV-Cre) TRN cells. **b**, Representative images showing YFP expression of the TRN Gad2^{+} (left) and PV^{+} (right) axon terminals within the PVT region. **c.** Top left: Schematic of the experimental approach for *ex vivo* whole-cell patch-clamp recordings of avTRN^{GAD2}-evoked oIPSCs in pPVT neurons. Bottom left: Representative recording from a pPVT cell with avTRN^{GAD2}-mediated oIPSC in ACSF (black), TTX (1 μM, red), 4AP (100 μM, green), and PTX (100 μM, yellow). Middle: Quantification of oIPSC amplitudes throughout an entire recording for a representative cell recorded in the pPVT. Right: Summary of oIPSC amplitudes for all pPVT cells. For some recordings, PTX was not bath applied. n=8 cells from 3 mice (PTX applied to 5 of 8 cells), repeated measures One-way ANOVA, $F_{(2,7)}$=12.67, P=0.0074. **d.** Left: Schematic of experimental approach for monosynaptic recordings of PL-evoked oEPSCs onto either pPVT or avTRN cells. Middle: Representative

recordings of PL-mediated oEPSCs in one pPVT cell (PL-PVT, red) and one avTRN cell (PL-TRN, blue) obtained from the same mouse in the presence of TTX and 4AP. Right: Quantification of oEPSC amplitudes for all pPVT and avTRN cells. PL-PVT n=15 cells from x mice; PL-TRN n=8 cells from 3 mice; Independent two-tailed *t*-test, $t_{(20)}$=4.689, ***P <0.0001. All anatomical experimented were repeated at least once, and similar results were obtained. Data are shown as mean ± s.e.m. Source data are provided as a Source Data file.

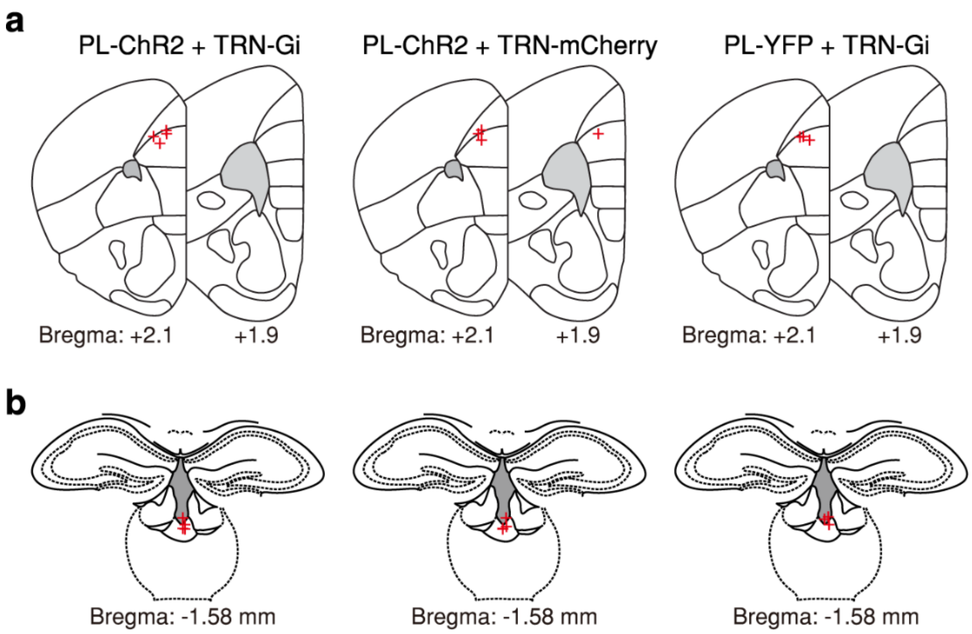

Supplementary Figure 3 - Optical fiber placement, related to Figure 3.

a-b, Optical fiber placement in PL (a) and PVT (b) regions (PL-ChR2 + TRN-Gi group, n=4 mice; PL-ChR2 + TRN-mCherry group, n=4 mice; PL-YFP + TRN-Gi group, n=3 mice).

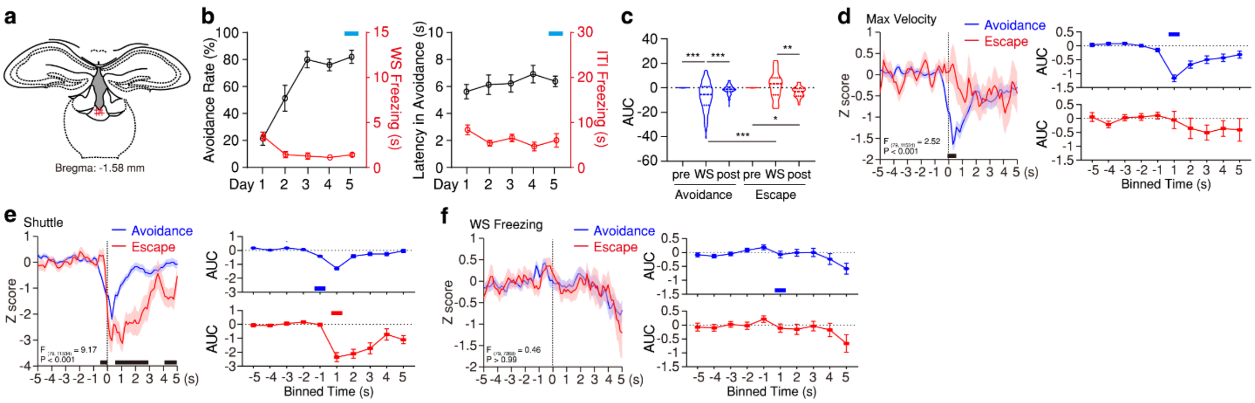

Supplementary Figure 4. Other data of fiber photometry experiment of the PL–PVT pathway, related to Figure 4.

a, Optical fiber placement in PVT region (n=5 mice). **b**, Left: Avoidance Rate and WS Freezing across training and imaging sessions (Highlighted with blue line). Right: Latency to avoid in avoidance trials and ITI Freezing across training and imaging sessions. **c**, Calcium signal AUC quantification for Avoidance and Escape trials. Mixed-effects model (REML) interactions $F_{(2, 296)}$=18.64, P<0.0001 (Avoidance, n=123 Trials; Escape, n=27 Trials). **d-f**, Left: Averaged calcium responses during WS maximal velocity (d), shuttle (e) and WS freezing (f) events for all Avoidance (blue) and Escape (red) trials. Mixed-effect model (REML) interactions reported in graphs. Max velocity: (Avoidance, n=122 Events; Escape, n=26 Events), Shuttle: (Avoidance, n=122 Events; Escape, n=26 Events) WS freeze: (Avoidance, n=67 Events; Escape, n=26 Events). Right: Calcium signal AUC quantification for each event in 1s bins, Mixed-effect model (REML) interactions reported in graphs. For all quantifications, multiple comparisons were conducted corrected by two-stage linear step-up procedure, black lines along x axis indicate significant changes reported between groups, and red or blue lines denote

the first significant change from the previous bin for within trial type comparisons. Data are shown as mean ± s.e.m. Source data are provided as a Source Data file.

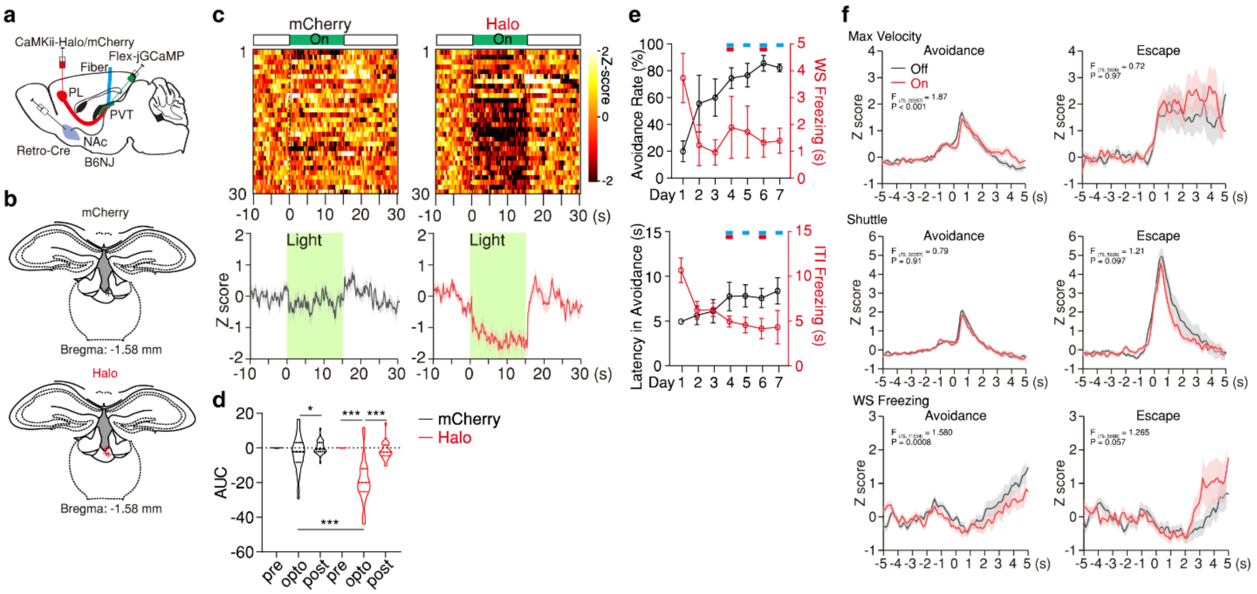

Supplementary Figure 5. PL input regulates the basal activity of NAc-projecting PVT neurons.

a, Schematic of the experimental approach for fiber photometry imaging of NAc-projecting pPVT cells and optogenetic inhibition of the PL-PVT pathway in naïve animals. **b**, Optic fiber placement in PVT region (n=3 mice per group). **c**, Top: Heatmaps of single trial calcium responses for control (mCherry) and Halo subjects. Bottom: Average calcium signal. **d**, Calcium signal AUC quantification for control and Halo subjects. Mixed-effects model (REML), $F_{(2,\,110)}$=28.64, P<0.0001 (n=30 Trials from 3 mice for each group). **e**, Top: Avoidance Rate and WS Freezing across training with imaging (blue) and opto (red) sessions. Bottom: Latency to avoid in avoidance trials and ITI Freezing across training and imaging and opto sessions. **f**, Averaged calcium responses for all Avoidance and Escape trials during WS maximal velocity (top), shuttle (middle), and WS freeze (right) events. Mixed-effects model (REML) interactions reported in corresponding graphs. Max velocity: Avoidance (Off n=144 Events from 3

mice, On n=141 Events); Escape, (Off n=36 Events, On n=37 Events). Shuttle: Avoidance, (Off n=144 Events, On n=141 Events); Escape, (Off n=36 Events, On n=37 Events). WS freeze: Avoidance, (Off n=74 Events, On n=74 Events); Escape, (Off n=32 Events, On n=42 Events). For all quantifications, multiple comparisons were conducted corrected by two-stage linear step-up procedure, black lines along x axis and asterisks denote significant changes reported between groups. Data are shown as mean ± s.e.m. Source data are provided as a Source Data file.

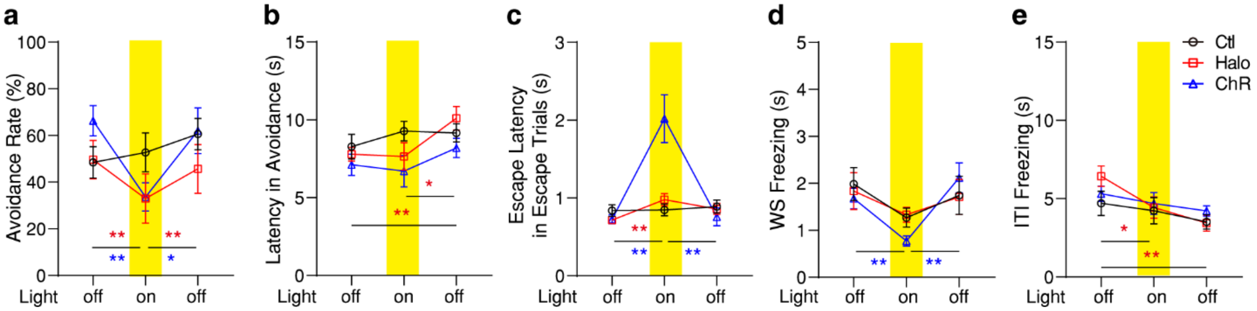

Supplementary Figure 6 - Raw data of optogenetic manipulations of the PL–PVT pathway, related to Figure 4.

a-e, Average avoidance rate (a), Latency to avoid (b), Latency to escape (c) and Freezing time during the WS (d) and ITI (e) across test sessions for each group. Two-way ANOVA (Ctl, n=11 mice; Halo, n=10 mice; ChR, n=10 mice). Interactions for Avoidance rate: $F_{(4, 56)}$=5.67, P=0.0007; Latency to avoid: $F_{(4, 53)}$=1.31, P=0.28; Latency to escape: $F_{(4, 56)}$ = 12.13, P < 0.0001; WS Freezing: $F_{(4, 56)}$ = 0.95, P = 0.44; ITI Freezing: $F_{(4, 56)}$ = 1.08, P = 0.38. For all quantifications, multiple comparisons were conducted corrected by two-stage linear step-up procedure. Asterisks denote where significance was found. Data are shown as mean ± s.e.m. Source data are provided as a Source Data file.

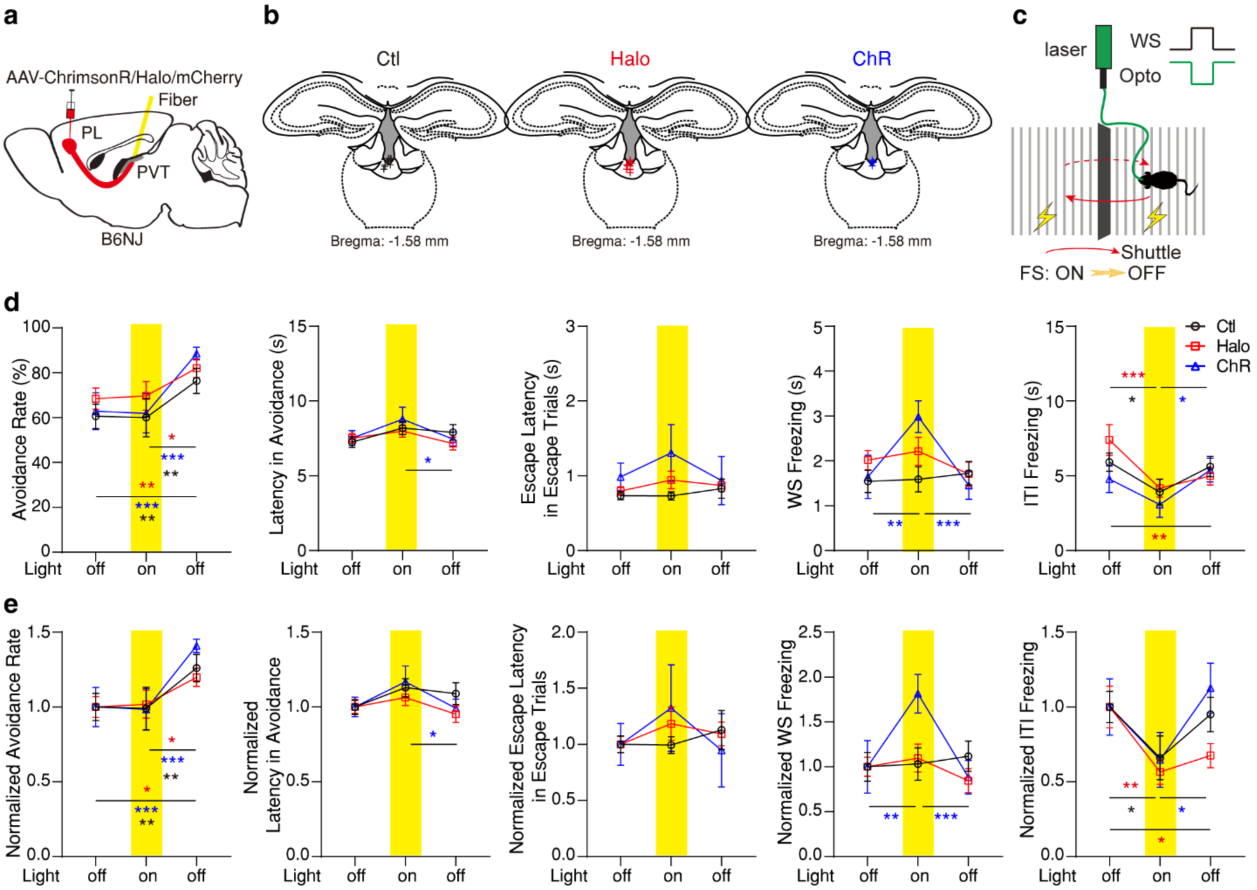

Supplementary Figure 7 – Optogenetic manipulation of PL-PVT pathway during the ITI has negligible effects on active avoidance behavior.

a, Viral strategy schematic for bidirectional optogenetic manipulations of PL-PVT circuit. **b**, Fiber placement in pPVT (Ctl, n=11 mice; Halo, n=13 mice; ChR, n=7 mice). **c**, 2AA task schematic. **d**, Group average avoidance rates, latencies to avoid, latencies to escape, and freezing times during the WS and ITI across test sessions. Two-way ANOVA interactions for: Avoidance rate: $F_{(4, 56)}$=0.97, P=0.43; Latency to avoid: $F_{(4, 56)}$=0.83, P=0.51; Latency to escape: $F_{(4, 55)}$=0.78, P=0.54; WS Freezing: $F_{(4, 56)}$=2.72, P=0.039; ITI Freezing: $F_{(4, 56)}$=1.362, P=0.26. **e**, Data normalized to first light-off session for each group taken from panel d. Two-way ANOVA multiple interactions for Avoidance

rate: $F_{(4, 56)}$=1.1, P=0.37; Latency to avoid: $F_{(4, 56)}$=0.82, P=0.52; Latency to escape: $F_{(4, 55)}$=0.74, P=0.57; WS Freezing: $F_{(4, 56)}$=3.07, P=0.023; ITI Freezing: $F_{(4, 56)}$=1.23, P=0.31. For all quantifications, multiple comparisons were conducted corrected by two-stage linear step-up procedure. Asterisks denote where significance was found. Data are shown as mean ± s.e.m. Source data are provided as a Source Data file.

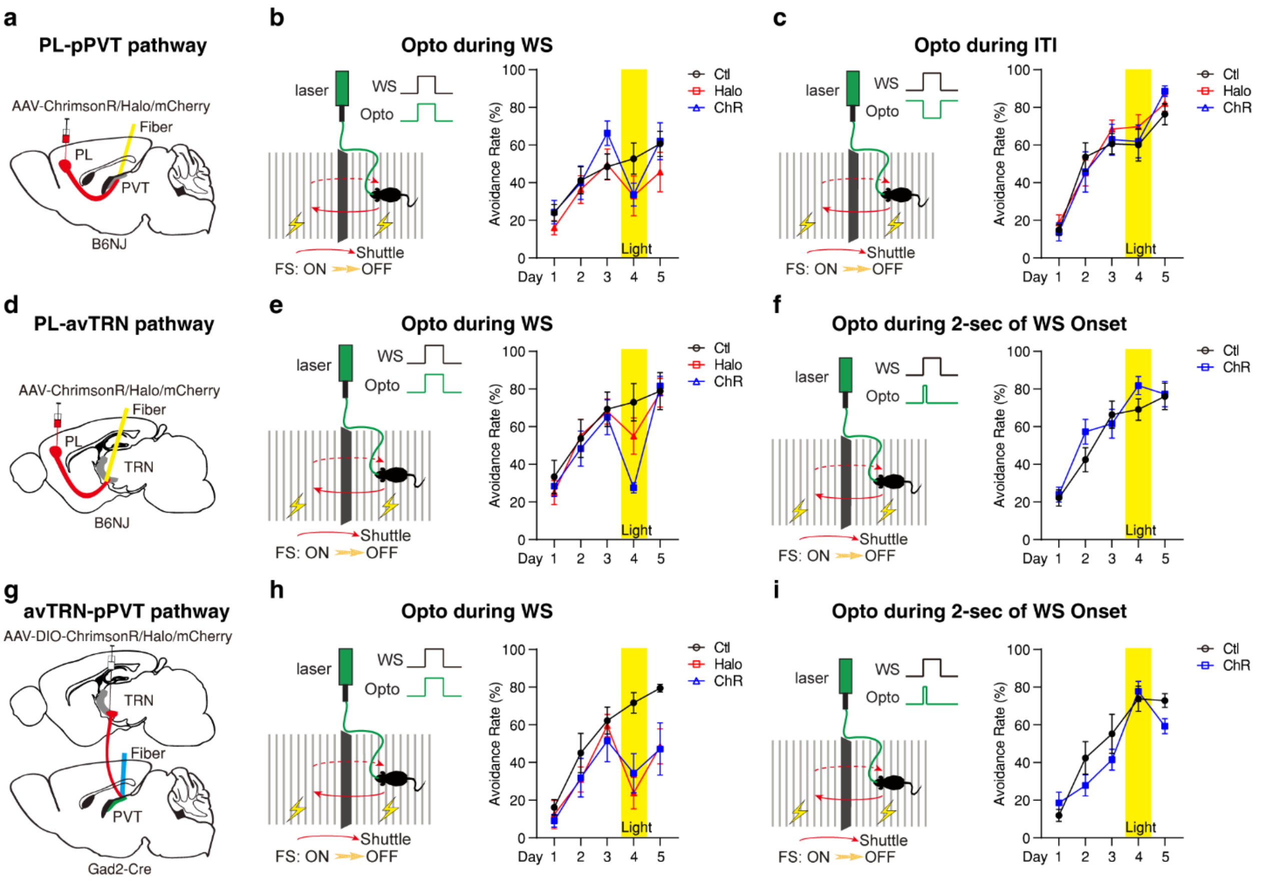

Supplementary Figure 8 – Avoidance learning curves of behavioral experiments, related to Figure 4-6, Supplementary Figure 6-7, 9, 10, 12-13 and 17.

a-c, Optogenetic manipulations of the PL-pPVT circuit; panel **b** is related to Figure 4e-f, Supplementary Figure 6 and 9; panel **c** is related to Supplementary Figure 7. **d-f**, Optogenetic manipulations of the PL-avTRN circuit; panel **e** is related to Figure 5h-i, Supplementary Figure 10g-h and Supplementary Figure 12b-c; panel **f** is related to Figure 5f-g, Supplementary Figure 10e-f and Supplementary Figure 12a. **g-i**, Optogenetic manipulations of the avTRN-pPVT circuit; panel **h** is related to Figure 6h-i, Supplementary Figure 13j-k, Supplementary Figure 14 and Supplementary Figure 17f-j; panel **i** is related to Figure 6f-g, Supplementary Figure 13h-i and Supplementary Figure

17a-e. Data are shown as mean ± s.e.m. Source data are provided as a Source Data file.

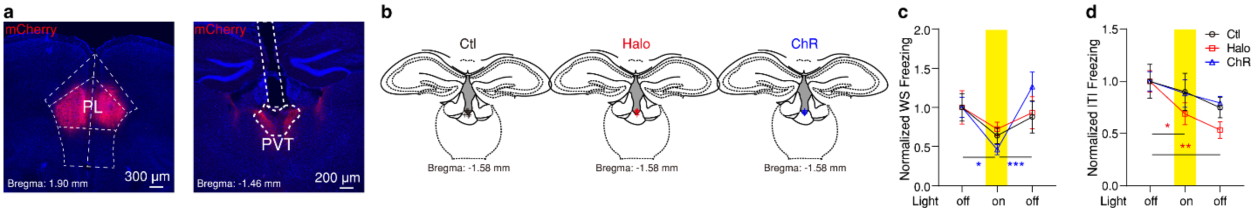

Supplementary Figure 9. Other data of optogenetic manipulations of PL–PVT projections, related to Figure 4.

a, Representative image of mCherry expression in the PL and PVT region. **b,** Optical fiber placement in PVT region (Ctl, n=11 mice; Halo, n=10 mice; ChR, n=10 mice). **c-d**, Group behavioral data across test sessions normalized to first light-off session for freezing times during WS (c) and ITI (d). Two-way ANOVA interactions for: WS Freezing: $F_{(4, 56)}$=1.28, P=0.29; ITI Freezing: $F_{(4, 56)}$=0.51, P=0.73. Data are shown as mean ± s.e.m. Source data are provided as a Source Data file.

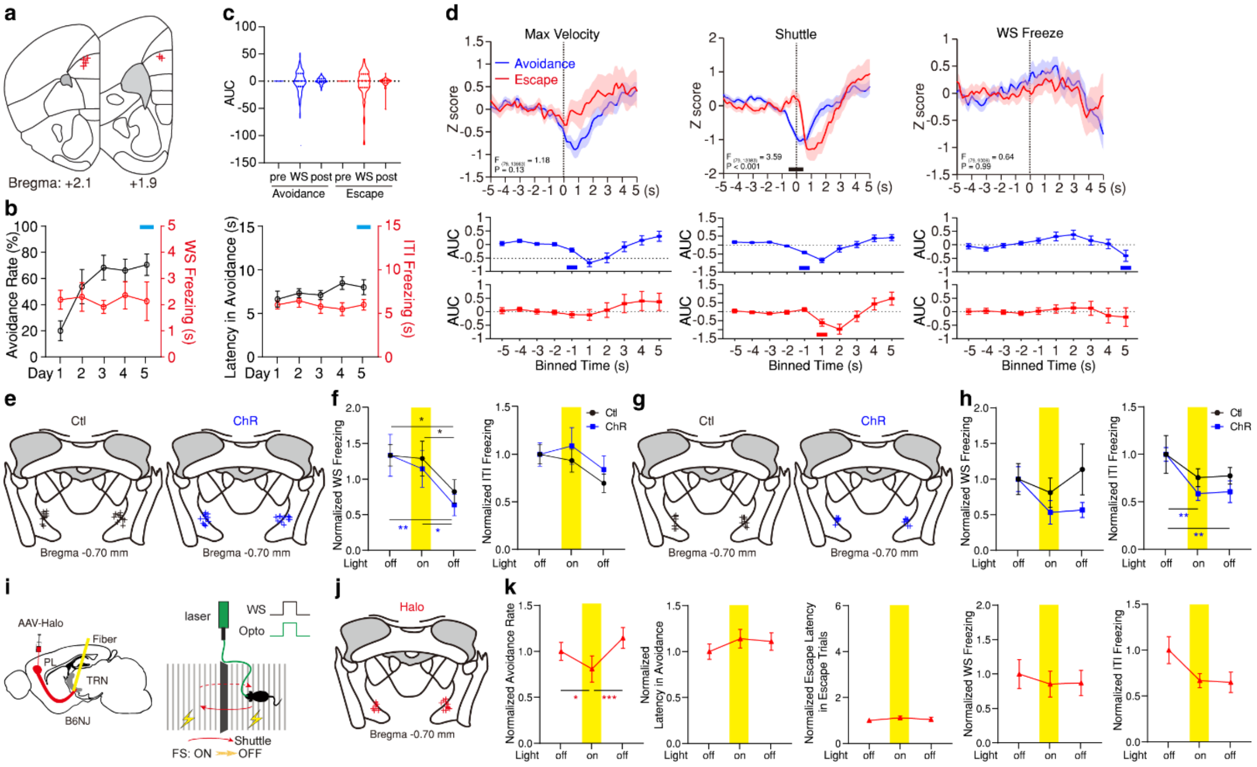

Supplementary Figure 10. Other data of fiber photometry and optogenetic experiments of PL–avTRN projections, related to Figure 5.

a, Optical fiber placement in PL, related to Fig. 5a-d (n=6 mice). **b**, Left: Avoidance Rate and WS Freezing across training and imaging session (blue line). Right: Latency to avoid in Avoidance trials and ITI Freezing across training and imaging session. **c**, Calcium signal AUC quantification for avoidance (blue) and escape (red) trials (n=128 Avoidance trials, 52 Escape trials). Mixed-effects model (REML), $F_{(2, 356)}$=0.51, P=0.60. **d**, Top: Averaged calcium responses during Max velocity (left, Avoidance, n=126 Events; Escape, n=53 Events), Shuttle (Middle) and WS freezing (right, Avoidance, n=64 Events; Escape, n=52 Events). Mixed-effects model REML interactions reported in graphs. Bottom: Calcium signal AUC quantification in 1s bins, Mixed-effects model (REML) interactions reported in graphs. **e**, Optical fiber placement in TRN region related

to Figure 5f-g (n=11 mice per group). **f**, Group behavioral data across test sessions normalized to first light-off session for freezing times during the WS (Left) and ITI (Right). Two-way ANOVA interactions reported in graphs. **g**, Optical fiber placement in TRN region related to Figure 5h-i (Ctl, n=9 mice; ChR, n=8 mice). **h**, Group behavioral data across test sessions normalized to first light-off session for freezing times during the WS (Left) and ITI (Right). Two-way ANOVA interactions reported in graphs. **i**, Left: Schematic of the viral vector strategy for optogenetic inhibition of PL-TRN circuit. Right: Schematic of the 2AA task. **j**, Optic fiber placement in TRN region (n=10 mice). **k**, Group behavioral data across test sessions normalized to to first light-off session for avoidance rate, latency to avoid, latency to escape, and freezing time during the WS and ITI (One-way ANOVA interactions reported in corresponding graphs). For all quantifications, multiple comparisons were conducted corrected by two-stage linear step-up procedure, black lines along x axis indicate significant changes reported between groups, and red or blue lines denote the first significant change from the previous bin for within trial type comparisons. Asterisks denote where significance was found. Data are shown as mean ± s.e.m. Source data are provided as a Source Data file.

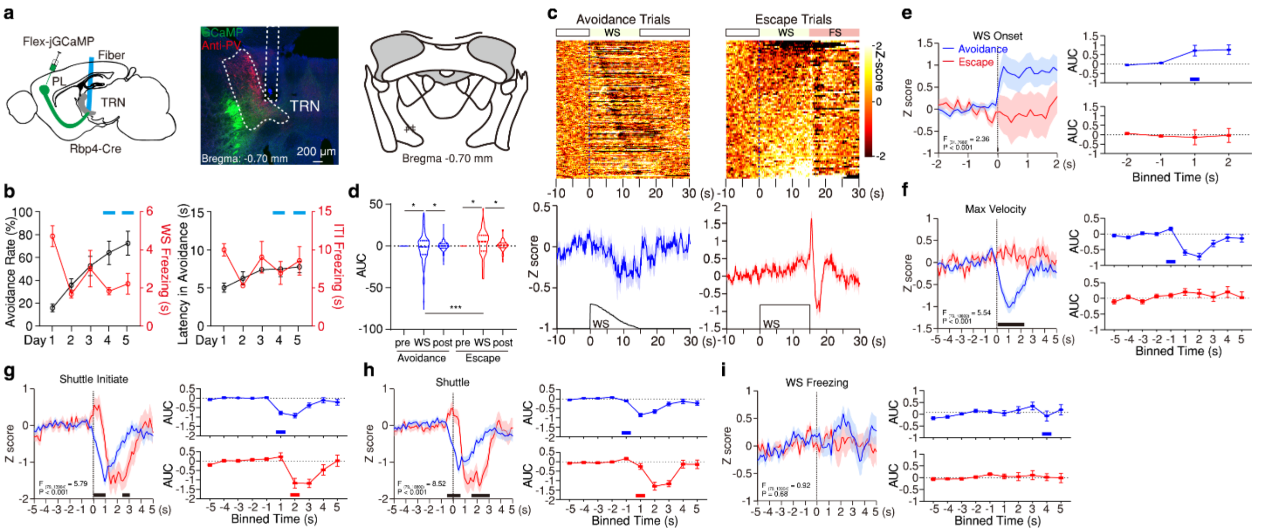

Supplementary Figure 11. The L5 PL–TRN pathway signals avoidance decisions.

a, Left: Schematic of the experimental approach for fiber photometry imaging of the axon terminals of PL Rbp4^{+} cells in the avTRN region. Middle: Representative images of GCaMP8s, PV expression, and optical fiber placement in the avTRN. Right, Optical fiber placement in the avTRN region (n=4 mice). **b**, Left: Avoidance Rate and WS Freezing across training and imaging sessions (Highlighted with blue line). Right: Latency to avoid in Avoidance trials and ITI Freezing across training and imaging sessions. **c**, Top: Heatmaps of calcium responses for Avoidance and Escape trials. Bottom: Average calcium signal and WS duration. **d**, Calcium signal AUC quantification for Avoidance and Escape trials. Mixed-effects model (REML), $F_{(2, 476)}$=6.51, P=0.0016, Avoidance, n=174 Trials; Escape, n=76 Trials; **e-i**, Left: Averaged calcium responses for Avoidance (blue) and Escape (red) trials during WS onset and REML comparisons for (e), Max velocity (f), Shuttle initiate (g), Shuttle (h) and WS freeze (i) events. For all quantifications multiple comparisons were conducted corrected by two-stage linear step-up procedure, black lines along x axis indicate significant changes reported

between groups, and red or blue lines denote the first significant change from the previous bin for within trial type comparisons. Asterisks denote where significance was found. Data are shown as mean ± s.e.m. Source data are provided as a Source Data file.

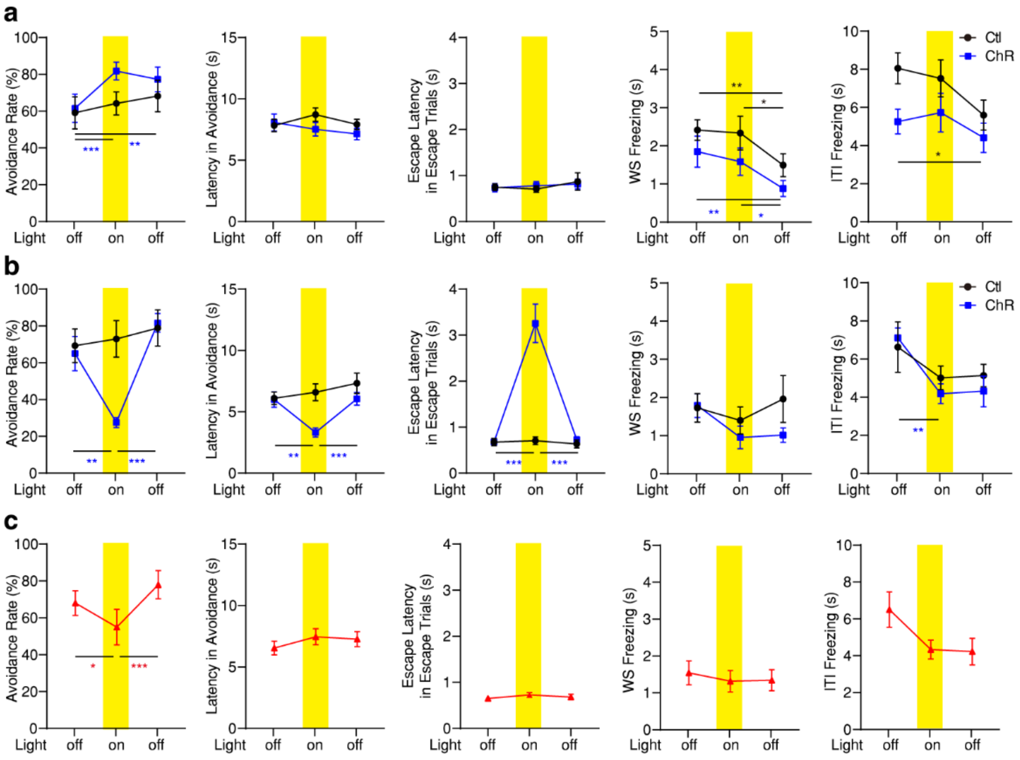

Supplementary Figure 12 - Raw data of optogenetic manipulations of the PL–avTRN circuit, related to Figure 5 and Supplementary Figure 10.

a, Related to Figure 5f-g and Supplementary Figure 9e-f. Average group Avoidance rates, latencies to avoid, latencies to escape, and freezing times during the WS and ITI across test sessions. **b**, Related to Figure 5h-i and Supplementary Figure 10g-h. Average avoidance rate, latency to avoid, latency to escape, and freezing time during the WS and ITI across test sessions for each group. (Ctl, n=9 mice; ChR, n = 8 mice). **c**, Related to Supplementary Figure 9i-k. Average avoidance rate, Latency to avoid, Latency to escape, and Freezing time during the WS and ITI across test sessions. Multiple comparisons corrected by two-stage linear step-up procedure of Benjamini, Krieger and Yekutieli were performed for all data in this figure. Data are shown as mean ± s.e.m. Source data are provided as a Source Data file.

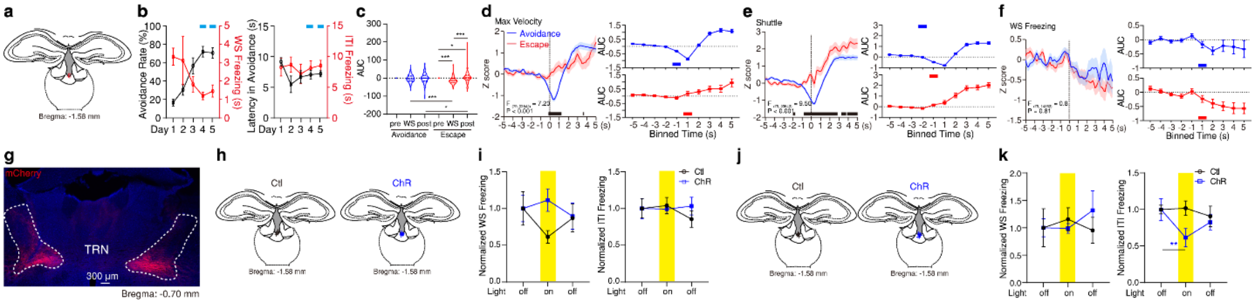

Supplementary Figure 13. Other data of fiber photometry and optogenetic experiments of avTRN –pPVT projections, related to Figure 6.

a, Optical fiber placement in PVT region (n=5 mice). **b**, Left: Avoidance Rate and WS Freezing across training and imaging sessions (blue line). Right: Latency to avoid in Avoidance trials and ITI Freezing across training and imaging sessions. **c**, Calcium signal AUC quantification for avoidance (blue) and escape (red) trials. **d-f**, Left: Averaged calcium responses during Max velocity (d), Shuttle (e) and WS freezing (f) events. Max velocity: Avoidance, n = 214 Events; Escape, n = 86 Events. Shuttle: Avoidance, n = 214 Events; Escape, n = 86 Events. WS Freezing: Avoidance, n = 104 Events; Escape, n = 86 Events. Right: Calcium signal AUC quantification in 1s bins for avoidance and escape trials. **g**, Representative image of mCherry expression in the TRN. **h**, Optic fiber placement in PVT region (Ctl, n = 7 mice; ChR, n = 9 mice), related to Figure 6f-g. **i**, Group behavioral data across test sessions normalized to first light-off session for freezing times during the WS and ITI. Two-way ANOVA interactions for WS Freezing: $F_{(2, 28)}$ = 1.81, P = 0.18; ITI Freezing: $F_{(2, 28)}$ = 0.61, P = 0.55. **j**, optical fiber placement in PVT region (Ctl, n = 6 mice; ChR, n = 7 mice), related to Figure 6h-i. **k**, Group behavioral data across test sessions normalized to first light-off session for

freezing time during the WS (Left) and ITI (Right). Two-way ANOVA multiple interactions for WS Freezing: $F_{(2, 22)}$ = 1.13, P = 0.34; ITI Freezing: $F_{(2, 22)}$ = 3.56, P = 0.046. For all quantifications multiple comparisons were conducted corrected by two-stage linear step-up procedure, black lines along x axis indicate significant changes reported between groups, and red or blue lines denote the first significant change from the previous bin for within trial type comparisons. Asterisks denote where significance was found. Data are shown as mean ± s.e.m. Source data are provided as a Source Data file.

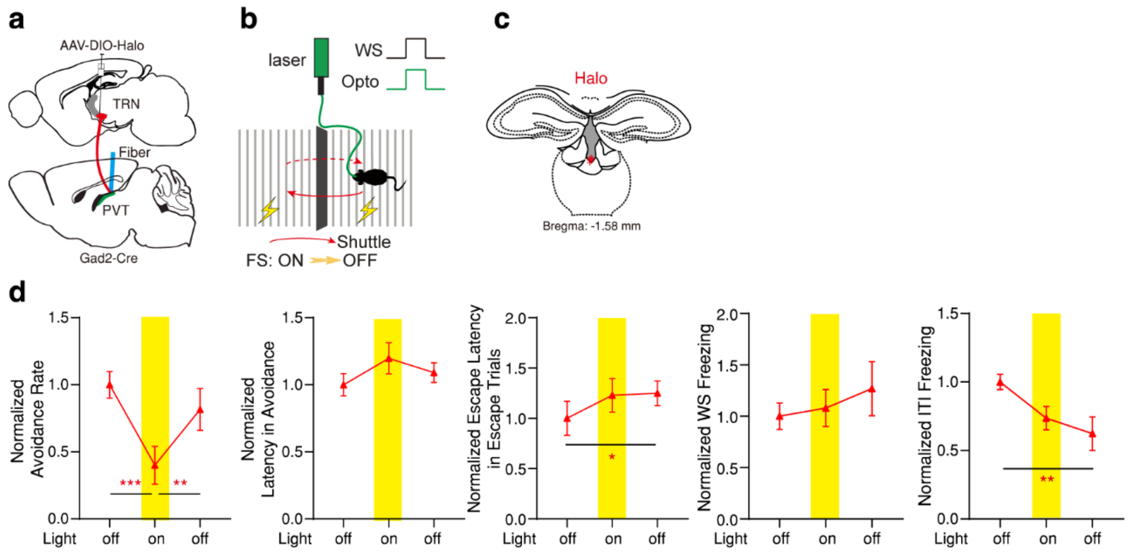

Supplementary Figure 14 - Optogenetic inhibition of the avTRN-pPVT circuit impairs active avoidance.

a, Schematic of the viral vector strategy for optogenetic inhibition of avTRN-pPVT circuit. **b**, Schematic of the 2AA task, the light is applied along with the WS. **c**, Optic fiber placement in PVT region (n = 7 mice). **d**, Group behavioral data across test sessions normalized to first light-off session for voidance rates, latencies to avoid, latencies to escape, freezing times during the WS and ITI. One-way ANOVA interactions reported for: Avoidance rate: $F_{(2, 12)}$=12.77, P=0.0011; Latency to avoid: $F_{(2, 12)}$=1.84, P=0.20; Latency to escape: $F_{(2, 12)}$=2.99, P=0.088; WS Freezing: $F_{(2, 12)}$=0.59, P=0.57; ITI Freezing: $F_{(2, 12)}$=5.01, P=0.026. Multiple comparisons corrected by two-stage linear step-up procedure of Benjamini, Krieger and Yekutieli were performed for all data in this figure. Asterisks denote where significant differences were found. Data are shown as mean ± s.e.m. Source data are provided as a Source Data file.

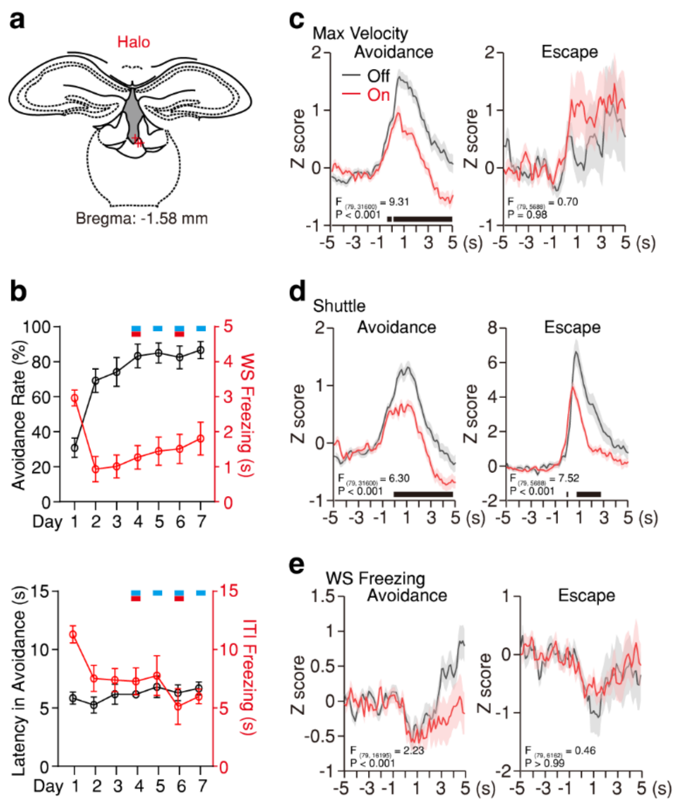

Supplementary Figure 15. Other data of fiber photometry experiment of the NAc-projecting PVT cells, related to Figure 7.

a, Optical fiber placement in PVT region (n=3 mice). **b**, Left: Avoidance Rate and WS Freezing across training with imaging (blue) and opto (red) sessions. Right: Latency to avoid in avoidance trials and ITI Freezing across training and imaging and opto sessions. **c-e**, Averaged calcium responses for Avoidance and Escape trials during Max velocity (c), Shuttle (d) and WS freeze (e) events. Max velocity: Avoidance, Off n=205 Events, On n=192 Events; Escape, Off n=34 Events, On n=40 Events. Shuttle: Avoidance, Off n=205 Events, On n=192 Events; Escape, Off n=34 Events, On n=40 Events. WS Freezing: Avoidance, Off n=116 Events, On n=91 Events; Escape, Off n=38 Events, On n=42 Events. Mixed-effects model (REML) interactions are reported on the

figures. Black thick lines along x axis indicate significant changes between Light On and Off averages from multiple comparisons corrected by two-stage linear step-up procedure. Asterisks denote where significance was found. Data are shown as mean ± s.e.m. Source data are provided as a Source Data file.

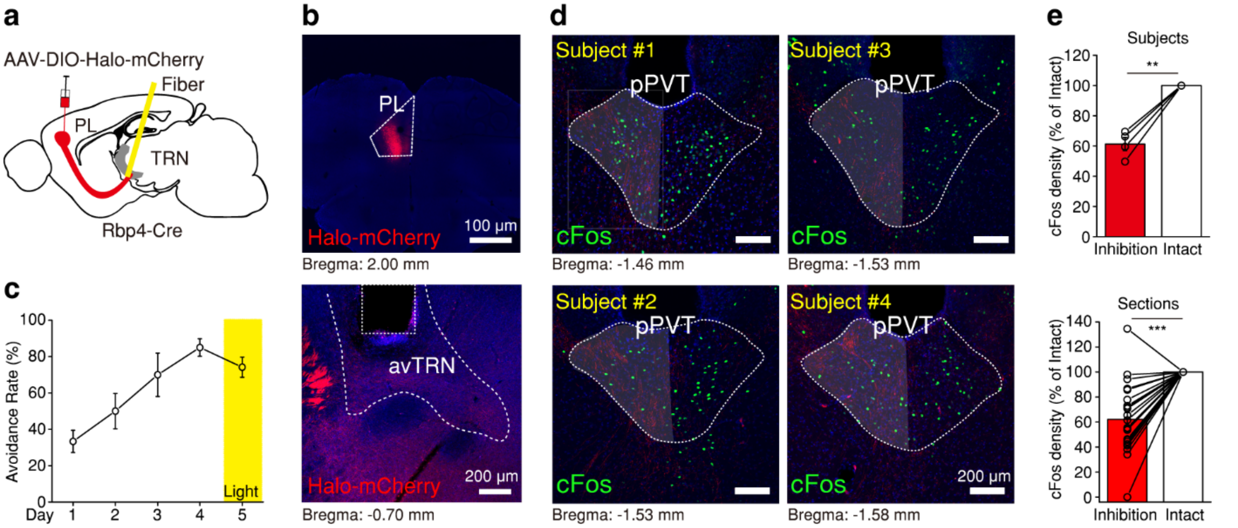

Supplementary Figure 16. PVT recruitment during the active avoidance task depends on PL-avTRN communication.

a, Schematic of the viral vector strategy used for optogenetic unilateral inhibition of PL–avTRN terminals in Rbp4-Cre mice. **b**, Representative images of Halo-mCherry expression in the PL and optical fiber placement in the avTRN region. **c**, Avoidance Rate across training and opto sessions (yellow background). **d**, Representative images of cFos immunostaining in the pPVT region. Left half of the pPVT in each image is highlighted as ipsilateral to the PL-avTRN inhibition. **e**, Quantification of the pPVT cells labeled with cFos antibody, separated by Inhibition and Intact half sides. Same data showed by subjects (Top) and sections (Bottom). For subjects, n=4 mice, paired Two-tailed *t*-Test, $t_{(3)}$=8.842, ***P*=0.0031. For sections, n=24 sections from 4 mice, paired Two-tailed *t*-Test, $t_{(23)}$=6.96, ****P*<0.0001. Data are shown as mean ± s.e.m. Source data are provided as a Source Data file.

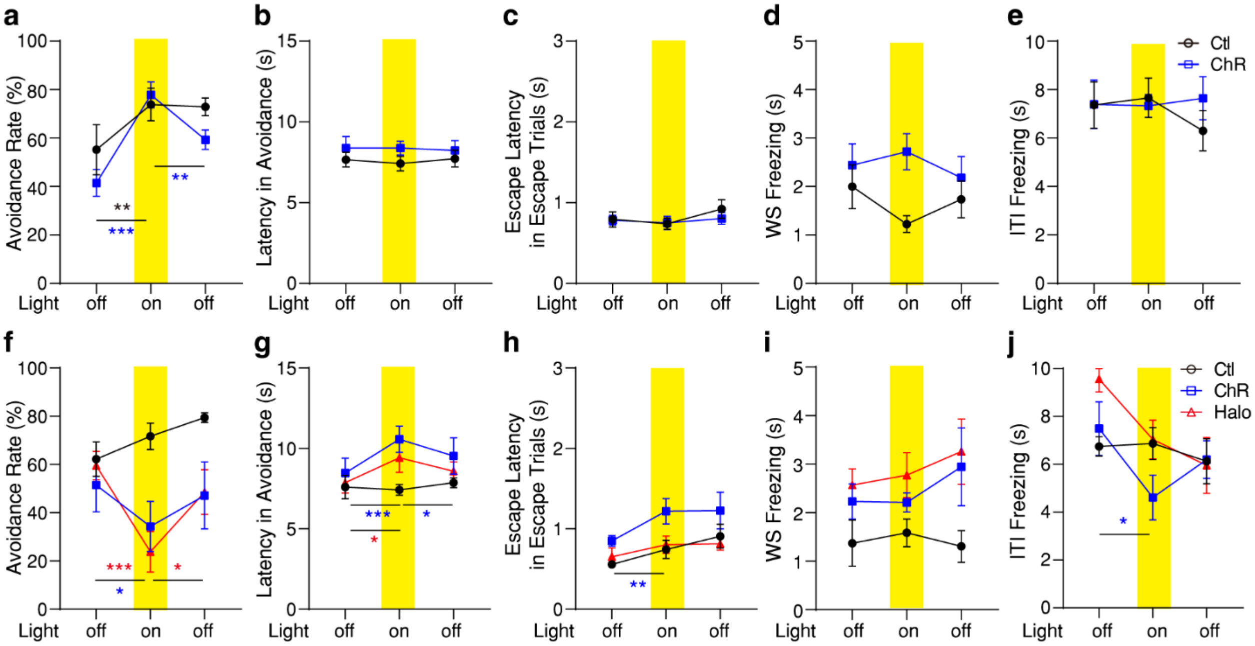

Supplementary Figure 17 - Raw data of optogenetic manipulations of the avTRN-PVT circuit, related to Figure 6, Supplementary Figure 13 and 14.

a-e, Related to Figure 6f-g and Supplementary Figure 13h-i. Average group avoidance rates (a), latencies to avoid (b), latencies to escape (c), and freezing times during the WS (d) and ITI (e) across test sessions. Two-way ANOVA interactions for Avoidance rate: $F_{(2, 28)}$ = 2.88, P = 0.073; Latency to avoid: $F_{(2, 28)}$ = 0.13, P = 0.88; Latency to escape: $F_{(2, 28)}$ = 0.96, P = 0.39; WS Freezing: $F_{(2, 28)}$ = 0.61, P = 0.23; ITI Freezing: $F_{(2, 28)}$ = 0.61, P = 0.55, (Ctl, n = 7 mice; ChR, n = 9 mice). **f-j**, Related to Figure 6h-i, Supplementary Figure 13j-k and Supplementary Figure 14. Average group avoidance rates (f), latencies to avoid (g), latencies to escape (h), and freezing times during the WS (i) and ITI (j) across test sessions. Two-way ANOVA interactions for Avoidance rate: $F_{(4, 34)}$ = 5.36, P = 0.0019; Latency to avoid: $F_{(4, 33)}$ = 2.18, P = 0.093; Latency to escape: $F_{(4, 34)}$ = 0.58, P = 0.68; WS Freezing: $F_{(4, 34)}$ = 0.51, P = 0.73; ITI Freezing: $F_{(4,}$

$_{34)}$ = 2.64, P = 0.051, (n = 6 mice; Halo, n = 7 mice; ChR, n = 7 mice). Multiple comparisons corrected by two-stage linear step-up procedure of Benjamini, Krieger and Yekutieli were performed for all data in this figure. Asterisks denote where significant differences were found. Data are shown as mean ± s.e.m. Source data are provided as a Source Data file.

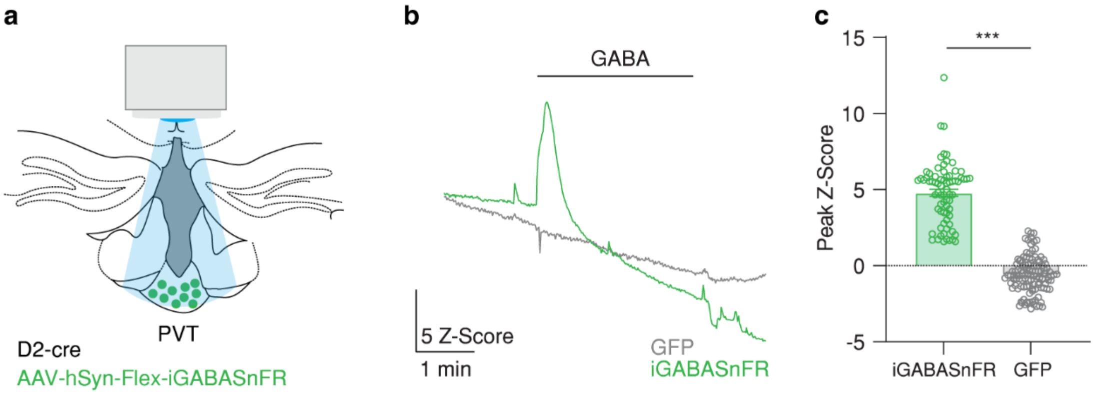

Supplementary Figure 18 – Single cell imaging validation of GABASnFR in acute slices.

a, Schematic of experimental design. **b,** Representative single cell imaging from a GABASnFR expressing cell (green) and a GFP expressing cell (grey) during bath application of GABA. **c,** Summary graphs of the peak z-score activity in response to GABA. GABASnFR n=73 cells from 3 mice, GFP n=115 cells from 3 mice, Independent two-tailed *t*-test, $t_{(186)}$=23.18, ****P*<0.0001. Data are shown as mean ± s.e.m. Source data are provided as a Source Data file.

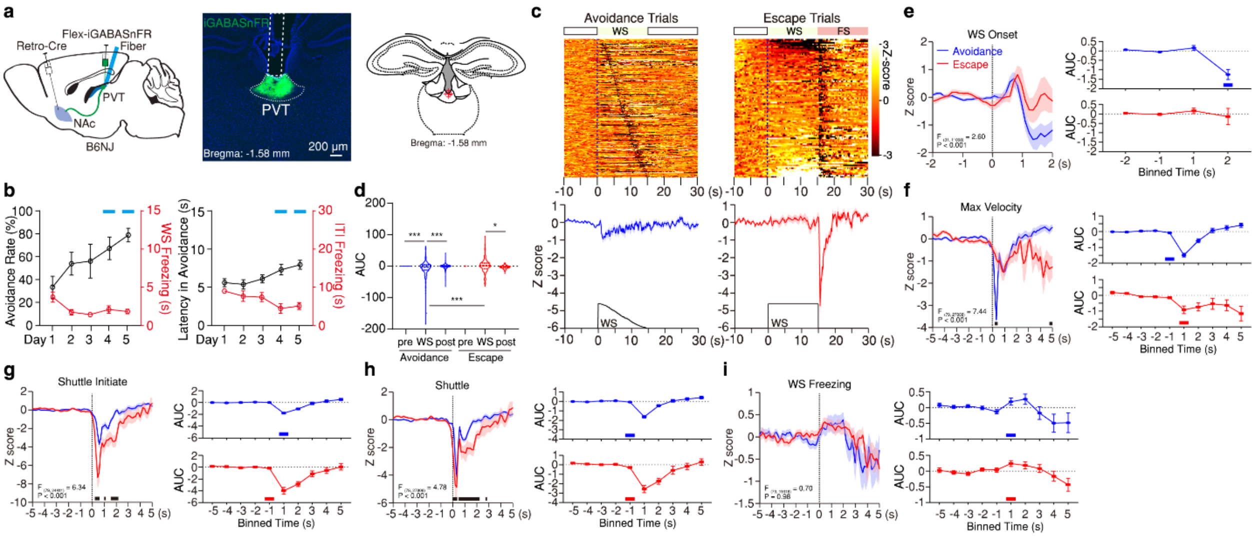

Supplementary 19. PVT extracellular GABA levels are modulated during active avoidance.

a, Left: Schematic of the experimental approach for fiber photometry imaging of GABA concentration in PVT neurons. Middle: Representative images of GABA sensor expression and fiber placement in PVT. Right, Fiber placement in PVT region (n=6 mice). **b**, Left: Avoidance Rate and WS Freezing across training and imaging sessions (blue line). Right: Latency to avoid and ITI Freezing across training and imaging sessions. **c**, Top: Heatmaps of calcium responses for avoidance and escape trials. Bottom: Average calcium signal and WS duration. **d**, Calcium signal AUC quantification for avoidance (blue) and escape (red) trials. Mixed-effects model (REML) interactions reported on the figures (Avoidance, n=263 Trials; Escape, n=97 Trials). **e-i**, Left: Averaged calcium responses for avoidance and escape trials during WS onset (e), Max velocity (f), Shuttle initiate (g), Shuttle (h) and WS freeze (i) events. WS onset: Avoidance, n=263 Events; Escape, n=97 Events. Max velocity: Avoidance, n=263 Events; Escape, n=96 Events. Shuttle initiate: Avoidance, n=221 Events; Escape, n=90

Events. Shuttle: Avoidance, n=263 Events; Escape, n=96 Events. WS Freezing: Avoidance, n=125 Events; Escape, n=119 Events. Right: Calcium signal AUC quantification for each event in 1s bins. For all quantifications multiple comparisons were conducted corrected by two-stage linear step-up procedure, black lines along x axis indicate significant changes reported between groups, and red or blue lines denote the first significant change from the previous bin for within trial type comparisons. Asterisks denote where significance was found. Data are shown as mean ± s.e.m. Source data are provided as a Source Data file.

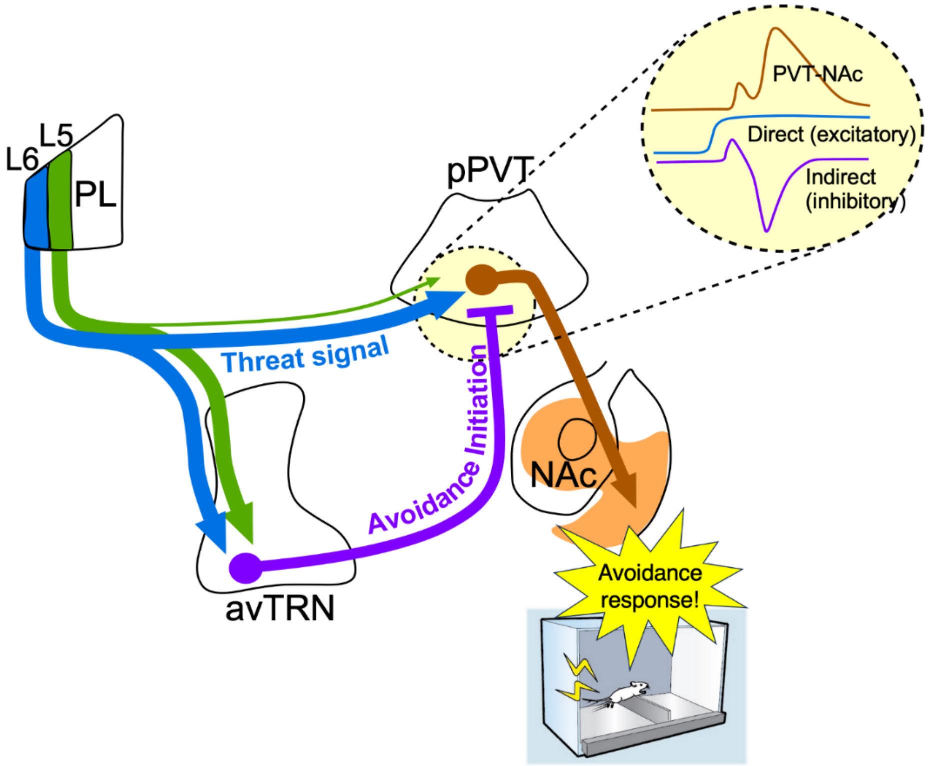

Supplementary Figure 20 – Proposed model of direct and indirect corticothalamic streams converging in the pPVT.

Schematic summarizing the findings of our study. PL shapes avoidance decisions via direct and indirect projections to the pPVT. While direct projections largely derived in L6 of PL control the spontaneous activity of PVT neurons, indirect projections via the inhibitory TRN shape task-related dynamics in the PVT. Both pathways work in conjunction to shape avoidance behavior, with the direct pathway priming the PVT (defensive state) and the indirect pathway timing behavioral engagement (disinhibition).
