# Supplementary material for: Convergent direct and indirect cortical streams shape avoidance decisions in mice via the midline thalamus
Source: Nat Commun. 2024 Aug 4;15:6598. doi: 10.1038/s41467-024-50941-6 (PMC11297946; doi:10.1038/s41467-024-50941-6)
Supplement: Supplementary file 3 — Reporting Summary [file 41467_2024_50941_MOESM3_ESM.pdf]

nature portfolio

Corresponding author(s): Mario A. Penzo

Last updated by author(s): Jul 11, 2024

Reporting Summary

Nature Portfolio wishes to improve the reproducibility of the work that we publish. This form provides structure for consistency and transparency in reporting. For further information on Nature Portfolio policies, see our Editorial Policies and the Editorial Policy Checklist.

Statistics

For all statistical analyses, confirm that the following items are present in the figure legend, table legend, main text, or Methods section.

n/a	Confirmed	
☐	☒	The exact sample size (*n*) for each experimental group/condition, given as a discrete number and unit of measurement
☐	☒	A statement on whether measurements were taken from distinct samples or whether the same sample was measured repeatedly
☐	☒	The statistical test(s) used AND whether they are one- or two-sided<br>*Only common tests should be described solely by name; describe more complex techniques in the Methods section.*
☒	☐	A description of all covariates tested
☐	☒	A description of any assumptions or corrections, such as tests of normality and adjustment for multiple comparisons
☐	☒	A full description of the statistical parameters including central tendency (e.g. means) or other basic estimates (e.g. regression coefficient) AND variation (e.g. standard deviation) or associated estimates of uncertainty (e.g. confidence intervals)
☐	☒	For null hypothesis testing, the test statistic (e.g. F, t, r) with confidence intervals, effect sizes, degrees of freedom and P value noted<br>*Give P values as exact values whenever suitable.*
☒	☐	For Bayesian analysis, information on the choice of priors and Markov chain Monte Carlo settings
☒	☐	For hierarchical and complex designs, identification of the appropriate level for tests and full reporting of outcomes
☒	☐	Estimates of effect sizes (e.g. Cohen's *d*, Pearson's *r*), indicating how they were calculated

Our web collection on statistics for biologists contains articles on many of the points above.

Software and code

Policy information about availability of computer code

Data collection	Fiber photometry data were obtained using Synapse version 92 (Tucker-Davis Technologies, FL). 2-way signaled active avoidance behavior was controlled and acquired using ANY-maze version 5 (Stoelting Co., IL). Electrophysiological recordings were collected using pClamp 10 (Molecular Devices, CA).
Data analysis	All data were analyzed using Origin Pro version 2016 and version 2018 (OriginLab Corp.) and GraphPad Prism version 8.0.1 (GraphPad Software). Electrophysiological data was analyzed using Igor Pro 9 (WaveMetrics, OR). Image analysis and cell counting were performed using ImageJ software (Fiji, version 1.52p). TopScan software (CleverSys) was used for video tracking of 2-way signaled active avoidance behavioral data. R Studio was used to analyze photometric signals. We used the R package version 2.2.2 (URL:https://CRAN.R-project.org/package=changepoint). R code used to analyze active avoidance behavior and photometric signal is available at the following repository: https://github.com/Penzolab/Data-analysis-of-Two-way-active-avoidance-task.git. (DOI: 10.5281/zenodo.12707790)

For manuscripts utilizing custom algorithms or software that are central to the research but not yet described in published literature, software must be made available to editors and reviewers. We strongly encourage code deposition in a community repository (e.g. GitHub). See the Nature Portfolio guidelines for submitting code & software for further information.

Data

Policy information about availability of data

All manuscripts must include a data availability statement. This statement should provide the following information, where applicable:

- Accession codes, unique identifiers, or web links for publicly available datasets
- A description of any restrictions on data availability
- For clinical datasets or third party data, please ensure that the statement adheres to our policy

The data used in this study has been put into a Source Data file and are available.

Research involving human participants, their data, or biological material

Policy information about studies with human participants or human data. See also policy information about sex, gender (identity/presentation), and sexual orientation and race, ethnicity and racism.

Reporting on sex and gender	*Use the terms sex (biological attribute) and gender (shaped by social and cultural circumstances) carefully in order to avoid confusing both terms. Indicate if findings apply to only one sex or gender; describe whether sex and gender were considered in study design; whether sex and/or gender was determined based on self-reporting or assigned and methods used. Provide in the source data disaggregated sex and gender data, where this information has been collected, and if consent has been obtained for sharing of individual-level data; provide overall numbers in this Reporting Summary. Please state if this information has not been collected. Report sex- and gender-based analyses where performed, justify reasons for lack of sex- and gender-based analysis.*
Reporting on race, ethnicity, or other socially relevant groupings	*Please specify the socially constructed or socially relevant categorization variable(s) used in your manuscript and explain why they were used. Please note that such variables should not be used as proxies for other socially constructed/relevant variables (for example, race/ethnicity should not be used as a proxy for socioeconomic status). Provide clear definitions of the relevant terms used, how they were provided (by the participants/respondents, the researchers, or third parties), and the method(s) used to classify people into the different categories (e.g. self-report, census or administrative data, social media data, etc.) Please provide details about how you controlled for confounding variables in your analyses.*
Population characteristics	*Describe the covariate-relevant population characteristics of the human research participants (e.g. age, genotypic information, past and current diagnosis and treatment categories). If you filled out the behavioural & social sciences study design questions and have nothing to add here, write "See above."*
Recruitment	*Describe how participants were recruited. Outline any potential self-selection bias or other biases that may be present and how these are likely to impact results.*
Ethics oversight	*Identify the organization(s) that approved the study protocol.*

Note that full information on the approval of the study protocol must also be provided in the manuscript.

Field-specific reporting

Please select the one below that is the best fit for your research. If you are not sure, read the appropriate sections before making your selection.

☒ Life sciences ☐ Behavioural & social sciences ☐ Ecological, evolutionary & environmental sciences

For a reference copy of the document with all sections, see nature.com/documents/nr-reporting-summary-flat.pdf

Life sciences study design

All studies must disclose on these points even when the disclosure is negative.

Sample size	The sample sizes used in our study, such as the numbers of animals, are typically the same or exceed those estimated by power analysis (power=0.80, α=0.05).
Data exclusions	Mice without correct targeting of optical fibers, tracers or vectors were excluded from this study. Access resistance was monitored throughout all recordings and recordings where access resistance increased more than 25% or above 30 MΩ were not included in analyses
Replication	All experiments were replicated at least once, and similar results were obtained.
Randomization	Animals were randomly allocated to the different experimental conditions reported in this study.
Blinding	For all behavior experiments, investigators were blinded to allocation during experiments. All 2AA photometric signals and behavioral performance were analyzed blinded. All RNAscope data were analyzed in a blinded manner

Reporting for specific materials, systems and methods

We require information from authors about some types of materials, experimental systems and methods used in many studies. Here, indicate whether each material, system or method listed is relevant to your study. If you are not sure if a list item applies to your research, read the appropriate section before selecting a response.

Materials & experimental systems

n/a	Involved in the study	
☐	☒	Antibodies
☒	☐	Eukaryotic cell lines
☒	☐	Palaeontology and archaeology
☐	☒	Animals and other organisms
☒	☐	Clinical data
☒	☐	Dual use research of concern
☒	☐	Plants

Methods

n/a	Involved in the study	
☒	☐	ChIP-seq
☒	☐	Flow cytometry
☒	☐	MRI-based neuroimaging

Antibodies

Antibodies used	Primary antibodies: anti-PV (1:1000, rabbit, Swant, PV 27) (overnight, at 4 °C); anti-CTB (1:10,000, goat, List Labs, Product No. 703) (48 h, at 4 °C). Secondary antibodies: Alexa Fluor-conjugated secondary antibodies (1:500, goat anti-mouse, Thermos Fisher Scientific A-11001; 1:500, goat anti-rabbit, Thermo Fisher Scientific A-21245).
Validation	The primary antibodies used in this study were validated by the manufacturer. For details, please refer to the manufacturer's website. The specificity of the secondary antibodies was established by lack of expression in tissue in which the primary antibodies were omitted.

Animals and other research organisms

Policy information about studies involving animals; ARRIVE guidelines recommended for reporting animal research, and Sex and Gender in Research

Laboratory animals	C57BL/6NJ (005304), Gad2-Cre (019022), and Pv-Cre (017320) were obtained from The Jackson Laboratory. Drd2-Cre (founder line ER44), Rbp4-Cre (founder line KL100), and Syt6-Cre (founder line KI148, 109, or 130) were obtained from GENSAT/MMRRC. Mice used in this study were group housed under a 12-h light/dark cycle (6:00–18:00 light), at temperatures of 70–74 °F and 40–65% humidity, with food and water available ad libitum. After surgery, mice were singly housed.
Wild animals	No wild animals were used in this study.
Reporting on sex	Both male and female mice 8–16 weeks of age were used for all experiments. We did not considered sex as a variable in this study. But similar results were observed for both sexes across experiments.
Field-collected samples	No field-collected samples were used in this study.
Ethics oversight	All procedures were performed in accordance with the Guide for the Care and Use of Laboratory Animals and were approved by the National Institute of Mental Health (NIMH) Animal Care and Use Committee.

Note that full information on the approval of the study protocol must also be provided in the manuscript.
